# Tmc Reliance Is Biased by the Hair Cell Subtype and Position Within the Ear

**DOI:** 10.3389/fcell.2020.570486

**Published:** 2021-01-07

**Authors:** Shaoyuan Zhu, Zongwei Chen, Haoming Wang, Brian M. McDermott

**Affiliations:** ^1^Department of Otolaryngology–Head and Neck Surgery, School of Medicine, Case Western Reserve University, Cleveland, OH, United States; ^2^Department of Biology, Case Western Reserve University, Cleveland, OH, United States; ^3^Department of Genetics and Genome Sciences, School of Medicine, Case Western Reserve University, Cleveland, OH, United States; ^4^Department of Neurosciences, School of Medicine, Case Western Reserve University, Cleveland, OH, United States

**Keywords:** hair cell, hearing, balance, mechanotransduction, Tmc, zebrafish

## Abstract

Hair cells are heterogenous, enabling varied roles in sensory systems. An emerging hypothesis is that the transmembrane channel-like (Tmc) proteins of the hair cell’s mechanotransduction apparatus vary within and between organs to permit encoding of different mechanical stimuli. Five anatomical variables that may coincide with different Tmc use by a hair cell within the ear are the containing organ, cell morphology, cell position within an organ, axis of best sensitivity for the cell, and the hair bundle’s orientation within this axis. Here, we test this hypothesis in the organs of the zebrafish ear using a suite of genetic mutations. Transgenesis and quantitative measurements demonstrate two morphologically distinct hair cell types in the central thickness of a vestibular organ, the lateral crista: short and tall. In contrast to what has been observed, we find that tall hair cells that lack Tmc1 generally have substantial reductions in mechanosensitivity. In short hair cells that lack Tmc2 isoforms, mechanotransduction is largely abated. However, hair cell Tmc dependencies are not absolute, and an exceptional class of short hair cell that depends on Tmc1 is present, termed a short hair cell erratic. To further test anatomical variables that may influence Tmc use, we map Tmc1 function in the saccule of mutant larvae that depend just on this Tmc protein to hear. We demonstrate that hair cells that use Tmc1 are found in the posterior region of the saccule, within a single axis of best sensitivity, and hair bundles with opposite orientations retain function. Overall, we determine that Tmc reliance in the ear is dependent on the organ, subtype of hair cell, position within the ear, and axis of best sensitivity.

## Introduction

In the ear, hair cells transduce mechanical stimuli for two senses: hearing and balance (Hudspeth, 1989). The soma of each of these epithelial cells presents a mechanosensitive hair bundle at its apical surface. Consisting of an array of stereocilia of graded lengths, the hair bundle is asymmetrical and is most sensitive along a single axis of best sensitivity (Shotwell et al., 1981). Both hearing and balance depend on hair cell heterogeneity (Eatock and Hurley, 2003; Kandel, 2013). The larval zebrafish ear contains three cristae, which have hair cells that encode accelerations from head rotations (Haddon and Lewis, 1996; Nicolson, 2005). In addition, the anterior (utricle) and posterior (saccule) maculae house hair cells to detect both linear accelerations for the sense of balance and vibrational stimuli for hearing.

Hair cells vary anatomically to accommodate an array of mechanical stimuli for hearing and balance. Hair cell variables include – but are not limited to – the organ that holds it, the morphology of the cell body (Sugihara and Furukawa, 1989), the position of the cell within the organ, and the axis of best sensitivity for the cell (Popper and Lu, 2000). Within the anamniotes, there are a few subtypes of hair cells that can differ in their morphologies and electrophysiological properties depending on the species and organ. The goldfish (*Carassius auratus*) saccule contains two differently sized hair cells: tall or gourd-like and short or eggplant shaped (Sugihara and Furukawa, 1989). In addition to shape differences, these cells contrast in their electrophysiological characteristics. The distribution of these differently sized cells is not homogenous: short hair cells tend to be found in the rostral saccule and both tall and short hair cells are found in the caudal saccule. Zebrafish saccular hair cells also have a stereotyped pattern in the direction their hair bundles face (Inoue et al., 2013). A similar pattern is seen in the mammalian saccule wherein hair cells are divided between two populations that have opposite hair bundle orientations (Deans, 2013). These hair cell populations are separated by a figurative boundary line, the line of polarity reversal. In the frog crista (*Rana pipiens*), there are morphologically distinct hair cells that are shaped like a club, cigar, or pear (Guth et al., 1994). These differently sized and shaped cells are distributed unevenly along the length and width of the crista. In contrast to the maculae, the hair bundles of the cristae are aligned in a single direction with the fluid motion of the semicircular cannels.

Transmembrane channel-like (Tmc) proteins have emerged as critical components of the hair cell’s mechanotransduction channel. In mammals, hearing and balance depend on TMC1 and TMC2 (Kawashima et al., 2011). In zebrafish, hearing uses Tmc1, Tmc2a, and Tmc2b (Chen et al., 2020). *Tmc2a* and *tmc2b* paralogs are the result of a whole-genome duplication that occurred in teleost fish between 300 and 450 million years ago (Taylor et al., 2001). Tmcs have several properties indicating that they are components of the mechanotransduction channel. In mammals and fish, Tmc proteins localize to the tips of stereocilia, the site of mechanotransduction (Kawashima et al., 2011; Maeda et al., 2014; Kurima et al., 2015; Chou et al., 2017; Mahendrasingam and Furness, 2019). There they are closely associated with other members of the mechanotransduction apparatus (Gleason et al., 2009; Maeda et al., 2014; Zhao et al., 2014; Erickson et al., 2017; Giese et al., 2017; Ge et al., 2018; Cunningham and Muller, 2019; Li et al., 2019; Pacentine and Nicolson, 2019; Cunningham et al., 2020). Finally, mutational, structural, and liposome-reconstitution studies support the hypothesis that TMCs are pore-forming subunits (Ballesteros et al., 2018; Pan et al., 2018; Jia et al., 2019).

Recently, we demonstrated that the zebrafish lateral line and maculae depend on different sets of Tmc proteins (Chou et al., 2017; Chen et al., 2020). In addition, we showed that hair bundle orientation can correlate with different Tmc use in a neuromast organ. Here, we examine the lateral cristae and posterior maculae of larval zebrafish to determine if five anatomical variables coincide with different Tmc use within the ear: organ, position within the organ, morphology, axis of hair bundle sensitivity, and hair bundle orientation within this axis.

## Results and Discussion

### Zebrafish Have Two Predominant Hair Cell Subtypes in the Central Thickness of the Lateral Crista: Tall and Short

**FIGURE 1:**
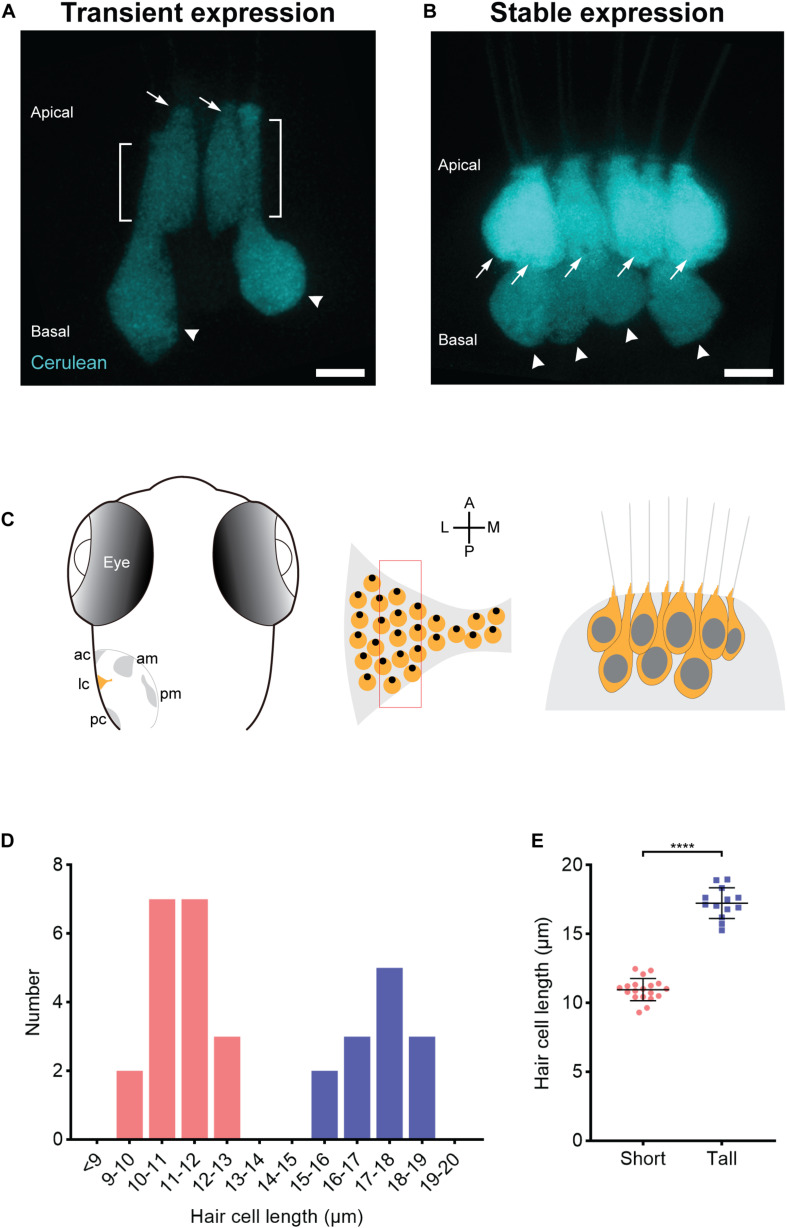
Two populations of morphologically distinct hair cells in the central thickness of the lateral crista of larval zebrafish. **(A,B)** Transient **(A)** and stable **(B)** transgenic expression of Cerulean fluorescent protein in the lateral crista reveals two morphologically distinct hair cells: short (arrows) and tall (arrowheads). The brackets mark the neck regions of tall hair cells. Scale bar = 5 μm. **(C)** Left panel, schematic of a dorsal view of a 7-dpf zebrafish ear, illustrating the five mechanosensory patches: the anterior crista (ac), lateral crista (lc), posterior crista (pc), anterior macula (am), and posterior macula (pm). Middle panel, enlarged view schematizing the hair cell arrangement in the lateral crista highlighted in yellow in the left panel. Each yellow circle represents a single hair cell, and the black dot within represents the position of the kinocilium (fonticulus). The red rectangle marks the section from which the hair cells are imaged and characterized. A, anterior; P, posterior; M, medial; L, lateral. Right panel, schematic of the two populations of hair cells (short and tall) in this section of the lateral crista based on imaging results. **(D)** The distribution of somata lengths of the lateral crista hair cells from 7-dpf zebrafish. *n* = 4. Pink bars represent short hair cells. Blue bars represent tall hair cells. **(E)** The somata lengths (mean ± SD) of the two groups of hair cells shown in panel **(D)**. ****P < 0.0001, unpaired *t*-test.

Since the sensory epithelia of goldfish and amphibians have hair cells with different morphologies, we examined the contours and dimensions of crista hair cells to quantitatively determine if these organs have differently shaped and sized hair cells or whether they are homogenous. To examine the crista for multiple hair cell subtypes based on morphology, we created somatic F0 mosaic transgenic zebrafish that express the fluorescent protein Cerulean under the control of the hair cell promoter *myosin 6b* (Cruz et al., 2015). This allowed us to identify sporadically labeled hair cells. Zebrafish at the larval stage were examined because they are optically clear, permitting scrutinization of hair cells *in vivo*. Using a confocal microscope, we imaged optical slices of the central thickness of the lateral crista because it is known that the lateral hair cells are developing in larvae (Haddon and Lewis, 1996), and the central region may contain the most mature population. Micrographs revealed two prevalent hair cell types: Tall hair cells with an elongated thin neck region and a nucleus close to the basal area of the cell and short hair cells that are stunted in comparison (Figures 1A,C). To quantitate length differences and determine spatial relationships between these cells, we generated a stable transgenic line, *Tg*(*myo6b:Cerulean*). In this section of the crista, tall and short hair cells are interdigitated (Figures 1B,C). Plots of hair cell lengths reveal two discernable populations (Figures 1D,E). The mean somata lengths of the short and tall hair cells are 11.0 ± 0.2 (mean ± SEM) μm and 17.1 ± 0.3 μm, respectively.

### Tall Hair Cells That Lack Tmc1 Display Defects in Mechanotransduction

**FIGURE 2:**
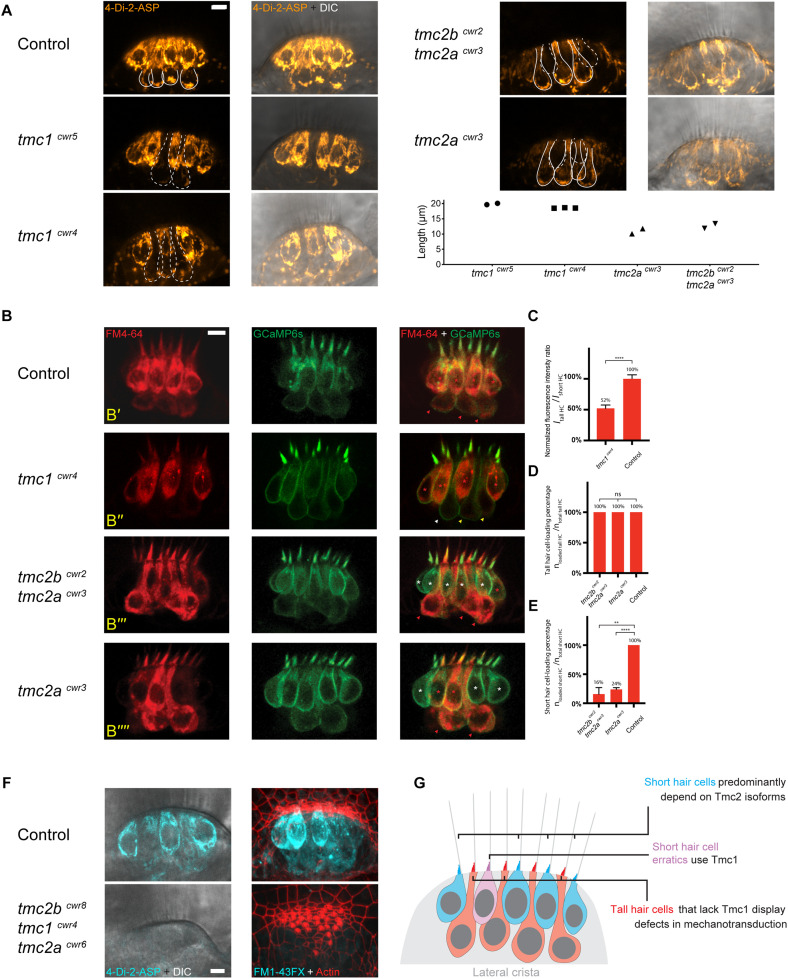
Tall and short hair cells of the lateral crista display biases for Tmc proteins in mechanotransduction. **(A)** Representative confocal images of 4-Di-2-ASP (orange) dye uptake assays showing differential dependencies of tall and short hair cells on Tmc proteins in the lateral cristae of 7-dpf larvae. Solid lines outline tall hair cells with 4-Di-2-ASP uptake in a wild-type sibling, a *tmc2a*^cwr3^ single mutant, and a *tmc2b*^cwr2^ *tmc2a*^cwr3^ double mutant. In each wild-type tall hair cell, there is an accumulation of dye near the cell’s base. The dashed lines outline tall hair cells with diminished 4-Di-2-ASP uptake in the *tmc1*^cwr5^ single mutant and the *tmc1*^cwr4^ single mutant or short hair cells with no or little 4-Di-2-ASP uptake in the *tmc2b*^cwr2^ *tmc2a*^cwr3^ double mutant and the *tmc2a*^cwr3^ single mutant. Graph of lengths of hair cells that have diminished 4-Di-2-ASP uptake. Each data point represents a single cell’s length and associated genotype. Cells associated with each data point are shown in confocal images with dashed outlines. **(B)** Representative confocal images of FM 4–64 (red) uptake assays for a heterozygous sibling control (*tmc2b*^cwr2/+^ *tmc2a*^cwr3/+^, **B’**), a *tmc1*^cwr4^ single mutant **(B”)**, a *tmc2b*^cwr2^ *tmc2a*^cwr3^ double mutant **(B”’)**, and a *tmc2a*^cwr3^ single mutant **(B””)**, each at 7-dpf. The lateral crista hair cells express GCaMP6s-CAAX (green), making each cell apparent. The red or white asterisks mark the short hair cells with or without FM4-64 loading, respectively. Red, yellow, or white arrowheads mark tall hair cells with normal, reduced, or no apparent FM4-64 dye loading, respectively. Red asterisk in panel **(B”’)** marks a short hair cell erratic that permits FM4-64 entry in the absence of Tmc2a and Tmc2b. **(C–E)** Graphs of normalized hair cell fluorescence intensity ratios (*I*_Tall HCs (hair cells)_/*I*_Short HCs_, **C**) and the percentages of hair cells that loaded with FM4-64 (*n*_loaded HCs_/*n*_total HCs_, **D,E**). **(C)** Two-tailed unpaired student’s *t*-test, ****P < 0.0001. **(D)** One-way ANOVA, *P* = 0.6076. **(E)** Two-tailed unpaired student’s *t*-test, ****P < 0.0001, **P = 0.0017. For the *tmc1*^cwr4^ single mutant, *n*_Tall HCs_ = 23, *n*_crista_ = 4. For the *tmc2b*^cwr2^ *tmc2a*^cwr3^ double mutant, *n*_Tall HCs_ = 15, *n*_short HCs_ = 28, *n*_crista_ = 3. For the *tmc2a*^cwr3^ single mutant, *n*_Tall HCs_ = 21, *n*_short HCs_ = 30, *n*_crista_ = 3. For the controls, *n*_Tall HCs_ = 27, *n*_short HCs_ = 40, *n*_crista_ = 4. **(F)** 4-Di-2-ASP (left, live) and FM1-43FX (right, fixed with hair bundles labeled by phalloidin) uptake assays demonstrate that mechanotransduction of lateral crista hair cells is absent in the *tmc2b*^cwr8^ *tmc1*^*cwr4*^ *tmc2a*^cwr6^ triple mutant, regardless of hair cell subtype. Control, *tmc2b*^cwr8/+^ *tmc1*^cwr4/+^ *tmc2a*^cwr6/+^. **(A,B,F)** Scale bar = 5 μm. **(G)** Model of Tmc dependencies in the central thickness of the lateral crista.

Since hair cells between the lateral line and the maculae of fish and between the maculae and the cochleae of mice (Kawashima et al., 2011) depend on different complements of Tmc proteins, we queried if these distinct hair cell subtypes rely on the same Tmc proteins or if their morphologies correlate with particular Tmc dependencies. To address this question, we implemented a genetic strategy using *tmc1* mutant zebrafish, *tmc1*^cwr5^, which lack the Tmc1 protein (Supplementary Tables S1, S2; Chen et al., 2020). To visually identify normal mechanosensitive hair cells versus those that may have lost or experienced a reduction in this defining property, we imaged the uptake of cationic fluorescent molecule 4-Di-2-ASP (Figure 2A), which passes through the hair cell’s mechanotransduction channel to act as an indicator of channel function (Gleason et al., 2009; Owens et al., 2009). After direct injection of 4-Di-2-ASP into the otocyst and subsequent confocal imaging, micrographs demonstrated that most hair cells in controls have functional mechanotransduction channels. However, in the *tmc1*^cwr5^ mutant, tall hair cells (length ∼19 μm) had significantly reduced labeling with 4-Di-2-ASP in the central thickness of the lateral crista, indicating that mechanotransduction is greatly diminished in these cells.

Because these outcomes disagree with recent findings by Smith et al. (2020), who stated that Tmc1 and Tmc2b are individually sufficient for MET function in tall hair cells and did not note a difference in uptake of FM dye in their *tmc1* mutant, we verified our findings using a second *tmc1* mutant strain, *tmc1*^cwr4^ (Supplementary Tables S1, S2; Chen et al., 2020). In the *tmc1*^cwr4^ mutant, tall hair cells in the central thickness of the lateral crista displayed a substantial reduction in uptake after 4-Di-2-ASP presentation (Figure 2A).

To further analyze the relationship between Tmc1 reliance and hair cell morphology, we applied a double-labeling scheme. Specifically, we generated *tmc1*^cwr4^ mutant larvae that express GCaMP6s-CAAX by outcrossing, injected their ears with fluorescent molecule FM4-64, and then imaged the hair cells of their cristae. GCaMP6s-CAAX is a fluorescent protein that labels the plasma membrane, defining the outline of each hair cell through illumination (Lukasz and Kindt, 2018). FM4-64 is a fluorescent molecule that passes through the transduction channel similarly to 4-Di-2-ASP (Meyers et al., 2003), and its emission spectra is distinguishable from that of GCaMP6s-CAAX. This semi-quantitative method detecting FM4-64 demonstrated that the *tmc1*^cwr4^ mutant that expressed GCaMP6s-CAAX contained tall hair cells with reduced mechanotransduction capacity. The flawed mechanotransduction was manifested by a reduction of ∼48% in the normalized fluorescence intensity ratio in the tall hair cell population (Figures 2B’,B”,C).

A potential explanation for the discrepancy between our findings and those of Smith et al. (2020) could be genetic in origin. Smith et al. (2020) used a zebrafish strain with a mutation in exon 3 of the *tmc1* gene. In our work, the *tmc1*^cwr4^ and *tmc1*^cwr5^ mutations are on exon 7 and exon 5, respectively. It is possible that exon skipping plays a role (Anderson et al., 2017), and exon 3 of *tmc1* is not as critical for the protein’s function in tall hair cells. Alternatively, Smith et al. (2020) may be imaging another region of the crista than the present study where hair cell Tmc dependencies are different. Nevertheless, tall hair cells in the central thickness of the lateral crista depend heavily on Tmc1 (Figure 2G).

### Short Hair Cells Predominantly Depend on Tmc2 Isoforms

Since tall hair cells are heavily dependent on Tmc1 for mechanotransduction, where short hair cells display no such reliance, we considered the possibility that short hair cells use Tmc2 isoforms, who are encoded by *tmc2a* and *tmc2b*. After injecting the larval ears of *tmc2b*^cwr2^ *tmc2a*^cwr3^ double mutants (Supplementary Tables S1, S2) with 4-Di-2-ASP and imaging optical sections of the central thicknesses of lateral cristae, we noted that most short hair cells (∼11 μm) do not permit passage of the fluorescent molecule, indicating that short hair cells require Tmc2 isoforms for mechanotransduction (Figures 2A,G). In contrast, in this double mutant, tall hair cell 4-Di-2-ASP labeling revealed no apparent phenotype.

Next, we looked to confirm the presence of unlabeled short hair cells using GCaMP6s-CAAX as a counter label in the double mutant. Specifically, we generated *tmc2b*^cwr2^ *tmc2a*^cwr3^ animals that express GCaMP6s-CAAX, and then imaged uptake of FM4-64 by hair cells of the lateral cristae. Similar to labeling with 4-Di-2-ASP, most short hair cells do not take up the FM4-64, contrasting with the tall hair cells, which permit entry of the fluorescent molecule (Figures 2B”’,D,E). Although most short hair cells lacking Tmc2 isoforms did not take up FM4-64, on occasion a short hair cell did permit entry of this fluorescent molecule. As these cells seemed out of place, we termed them short hair cell erratics (Figure 2B”’, bottom, red asterisk), borrowing from the geology term for rocks that appear incongruous with the local bedrock.

Because *tmc2a* mRNA is detectable in the crista by *in situ* hybridization, where *tmc2b* mRNA is not (Maeda et al., 2014), we aimed to determine if removal of Tmc2a alone could perturb mechanotransduction by short hair cells. Thus, we carried out 4-Di-2-ASP uptake studies on hair cells in *tmc2a*^cwr3^ single mutant zebrafish. Similar to the double mutant, the *tmc2a*^cwr3^ single mutant predominantly lacked 4-Di-2-ASP uptake by short hair cells (Figure 2A). Comparable results were obtained with FM4-64 injections into the ears of the *tmc2a*^cwr3^ single mutant that expresses GCaMP6s-CAAX (Figures 2B””,D,E). These findings confirm that short hair cells mainly depend on Tmc2 isoforms, and Tmc2a plays a significant role in these cells.

We hypothesized that the short hair cell erratics, who permit mechanotransduction despite being devoid of Tmc2 isoforms, are in fact dependent on Tmc1. To test this, we carried out 4-Di-2-ASP uptake studies on the *tmc2b*^cwr8^ *tmc1^cwr4^ tmc2a^cwr6^* triple mutant (Chen et al., 2020; Supplementary Tables S1, S2). None of the lateral crista hair cells permitted 4-Di-2-ASP entry, indicating that short hair cell erratics of the lateral crista are dependent on Tmc1 (Figure 2F). We confirmed these results using FM1-43FX, a fluorescent molecule that enters through the mechanotransduction channel (Gale et al., 2001; Meyers et al., 2003; Chou et al., 2017; Figure 2F). In all, these findings show that hair cell morphology coincides with a bias for specific Tmc use within the ear, but the relationship of a particular Tmc to a morphology need not be absolute.

### Hair Cell Position and Axis of Best Sensitivity Coincide With Tmc Dependencies in the Saccule

Next, we considered whether four hair cell anatomical variables – as they relate to Tmc dependencies – play a role in hearing: organ, position within an organ, axis of best sensitivity in which the hair bundle responds, and the hair bundle’s orientation within this axis. More specifically, a hair cell’s dependency on a particular Tmc may be restricted to one hair bundle orientation within an axis of best sensitivity (asymmetric model), or it may be free of this constraint (symmetric model) (Supplementary Figure S1). We explored Tmc dependencies in the saccule, which is considered the major hearing organ in the larval zebrafish ear (Inoue et al., 2013) and offers a sensory epithelium where the hair bundle orientations can be mapped and correlated to function.

**FIGURE 3:**
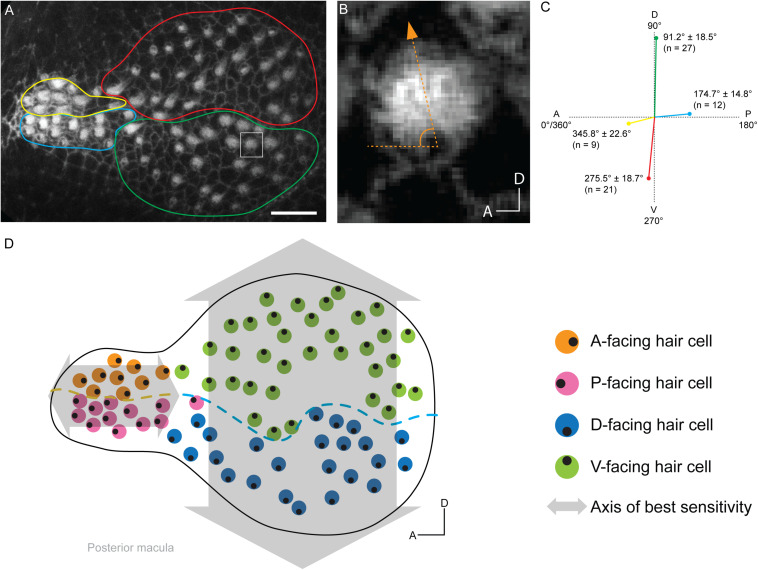
Map of hair bundle polarities in the posterior macula of a 7-dpf larva. **(A)** Confocal image of a single posterior macula labeled with phalloidin. Circumscribed regions contain groups of hair cells with particular hair bundle polarities: yellow – anterior, blue – posterior, red – ventral, and green – dorsal. **(B)** Enlarged view of a hair bundle in square in panel **(A)**. The hair bundles angle of deviation from the A-P axis is depicted. **(C)** Posterior macula hair bundle polarities and associated hair cell numbers. Each colored line length represents the number of hair cells with that orientation in each group from panel **(A)**. Each line orientation is the mean deviation from the A-P axis ± SD. **(D)** Map of hair bundle polarities in the posterior macula from panel **(A)**. Each color circle represents an individual hair cell, and the black dot within represents the position of the kinocilium, which reflects bundle polarity. Lines of polarity reversal are shown in the bulbus (blue dashed line) and narrow region (yellow dashed line) for D-V-facing and A-P-facing hair cells, respectively. Axes of best sensitivity are depicted by gray arrows.

To explore these four hair cell anatomical variables and their relationship to Tmc dependencies in the saccule, we took a two-step approach. We mapped the hair bundle orientations of the 7-dpf saccule of the wild-type *Tübingen* (*Tü*) strain by labeling with fluorophore-coupled phalloidin and then overlaid hair cell Tmc dependencies onto this organ. The first step was done in quadruplicate and a representative map is displayed in Figure 3. The map of the posterior macula revealed a stereotyped organization of hair bundles similar to those of 5-dpf animals (Inoue et al., 2013), showing that during this developmental time period the hair bundle orientations are not greatly altered. Overall, the organ is light bulb shaped (Figures 3A,D). There is an anterior narrowing where the dorsal hair cells tend to face anteriorly, but the ventral cells face posteriorly – establishing a single anterior–posterior axis of best sensitivity (Figures 3A,D). Hair bundles in the bulbous region have two orientations: the dorsal hair cells face ventrally, and the ventral hair cells face dorsally, yielding a single dorsal-ventral axis of best sensitivity (Figures 3B–D). There seems to be a transition area between the narrow region and the bulbous region that has hair bundles that may be in the process of orienting to the major axes of best sensitivity (Figure 3A,D). Overall, we show that 7-dpf larvae have two major axes of best sensitivity that are perpendicular to each other, and run parallel to those of the lateral line (Lopez-Schier et al., 2004; Chou et al., 2017), suggesting the paramount importance of detecting these directions for larval survival. Interestingly, the adult zebrafish saccule and those of goldfish and carp have just one axis of best sensitivity (Platt, 1993), contrasting with those of otophysan fishes who have two (Popper and Lu, 2000). Thus, the larval zebrafish saccular architecture – with respect to hair bundle orientations – is more similar to that of the latter superorder than it is to adult zebrafish.

Using this map, we explored if hair cell anatomical variables – either position of the hair cell, axis of best sensitivity, or a hair bundle’s orientation within this axis – play a role in Tmc dependencies in the ear. Alternatively, these variables may not be relevant and Tmc dependencies in the maculae may be organized by a different set of criteria or even randomly. To address this question, we used the *tmc2b*^cwr2^ *tmc2a*^cwr3^ double mutant larvae for a number of favorable properties that it offers. One, hair cell mechanotransduction in this fish strain is dependent on just one Tmc protein, Tmc1, eliminating the chances of complex Tmc interactions that could complicate the interpretation of results. Two, the hair cells that still function are in the posterior macula, but functional hair cells have not been mapped with high resolution (Chen et al., 2020; Smith et al., 2020). Three, this mutant can hear. Its capacity to do so is limited, but the presence of Tmc1 is able to mediate a C-start response to vibrational stimuli (Chen et al., 2020).

**FIGURE 4:**
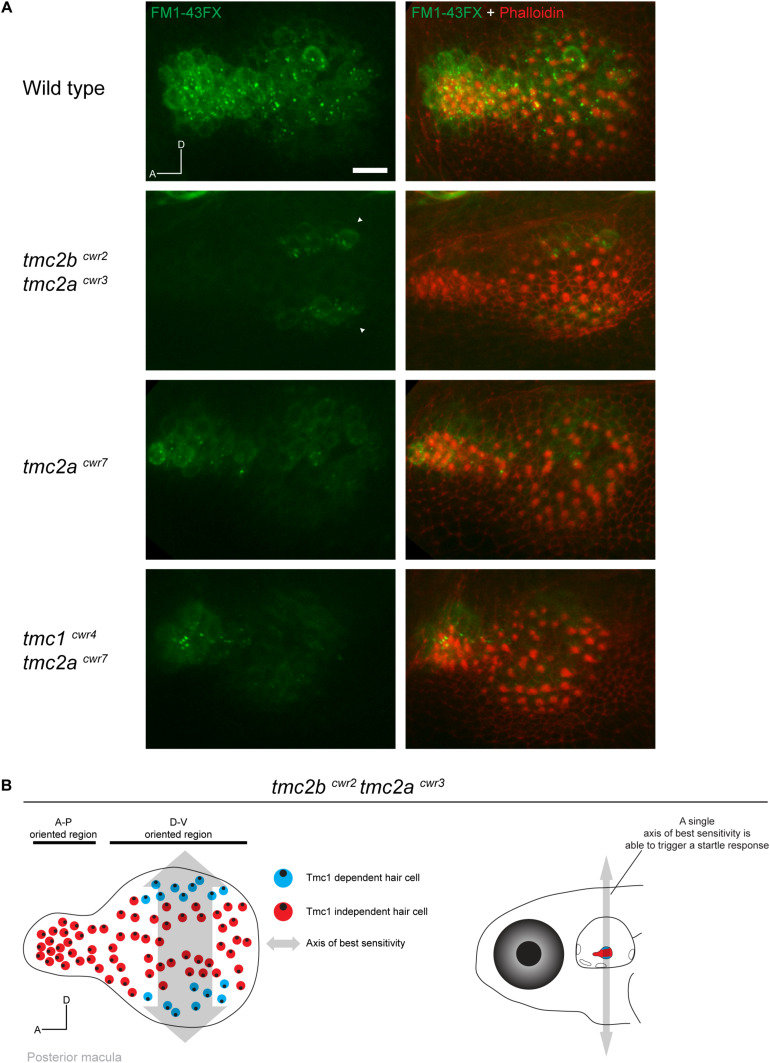
Hair cells of the posterior macula are differentially dependent on Tmc proteins. **(A)** Representative images of FM1-43FX (green) uptake in posterior maculae of wild-type and mutant larvae at 7 dpf. Hair bundles are labeled with phalloidin (red). In a wild-type macula, passage of FM1-43FX is observed in all hair cells. In the *tmc2b*^cwr2^ *tmc2a*^cwr3^ double mutant, FM1-43FX uptake is limited to two symmetrical stripes of hair cells located toward the posterior of the macula at the dorsal and ventral poles (arrowheads). In contrast, the *tmc2a*^cwr7^ single mutant or *tmc1*^cwr4^ *tmc2a*^cwr7^ double mutant hair cells do not reveal a pattern similar to the *tmc2b*^cwr2^ *tmc2a*^cwr3^ double mutant. A, anterior; D, dorsal. Scale bar = 10 μm. Similar patterns of saccular uptake were observed for five larvae for each mutant type and control. **(B)** (Left) Map of hair cell positions and hair bundle polarities in relation to Tmc1 use within the posterior macula based on the *tmc2b*^cwr2^ *tmc2a*^cwr3^ double mutant. Each color circle (red or blue) represents an individual hair cell, and the black dot within represents the position of the kinocilium, which reflects bundle polarity. Double-headed arrow indicates axis of best sensitivity preserved in the *tmc2b*^cwr2^ *tmc2a*^cwr3^ double mutant. (Right) Schematized zebrafish head depicting just one axis of best sensitivity (dorsal-ventral) able to elicit a startle response.

To evaluate the potential roles of the anatomical variables, we injected FM1-43FX into the ears of mutant zebrafish and labeled the animals with phalloidin to reveal hair bundle orientations and FM1-43FX uptake. High-resolution confocal images revealed that Tmc1-dependent transduction in the larval saccule is limited to just ∼10–18 hair cells. These Tmc1-dependent hair cells were highly constrained, located in the bulged end of the lightbulb-shaped organ in two symmetrical stripes (Figures 4A,B). The Tmc1-dependent stripes are located at opposite poles of the organ, dorsal and ventral. Each stripe is approximately two hair cells in thickness. All Tmc1-dependent hair cells fall within a unitary axis of best sensitivity, dorsal-ventral, and face both directions, preserving symmetry. This pattern of uptake is not observed in the *tmc2a*^cwr7^ single mutant or *tmc1^cwr4^ tmc2a^cwr7^* double mutant, indicating it is a pattern specific to Tmc1 (Figure 4A). These findings reveal a number of rules about the geometrical nature of Tmc1 dependencies. One, position is a major factor in determining Tmc1 dependence because only cells in the peripherally positioned stripes use this protein. Two, Tmc1-dependence falls within a single axis of best sensitivity, dorsal-ventral. Three, not all hair cells depend on Tmc1 in this axis, so it cannot be the only determining factor of Tmc1 dependence. Four, since Tmc1-dependent hair cells have opposite hair bundle orientations, a single hair bundle direction does not govern Tmc1 reliance, thus, satisfying the symmetric model of Tmc1 dependence (Supplementary Figure S1).

## Conclusion

Tmc proteins have emerged as mechanotransduction channel components of hair cells. The fundamental rules of how members of this small family of proteins are paired with hair cells within the crista and saccule for the inception of hearing and balance are poorly understood. We have investigated which, if any, of five hair cell anatomical variables coincide with different Tmc use within the ear: organ, position within the organ, morphology, axis of hair bundle sensitivity, and hair bundle orientation within this axis. First, we demonstrated that the zebrafish lateral crista has tall and short hair cells that have mean lengths of ∼17 and ∼11 μm, respectively. This finding demonstrates that fish have more than one hair cell type in the crista to detect head rotations. Moreover, using a suite of genetic mutations, we showed that tall hair cells that lack Tmc1 display defects in mechanotransduction, and short hair cells predominately depend on Tmc2 isoforms. However, there are rare short hair cells that do not follow these rules. Termed short hair cell erratics, these less frequently observed cells of the central thickness of the lateral crista use Tmc1. In all, these findings suggest that there are functional differences between the tall and short hair cells at the level of mechanotransduction.

The role of short hair cell erratics is unclear. They may be maturing hair cells that are transitioning between different Tmcs during the course of development, similar to what has been observed in mice (Kawashima et al., 2011). On the other hand, short hair cell erratics may be mature, functional hair cells that need to have a different Tmc complement than the similarly sized majority and contribute specifically to detecting rotational accelerations by adding diversity to the mechanotransduction response of the whole crista. The particular differences that may be instilled into mechanotransduction by each Tmc or each Tmc combination is yet unknown. However, it is clear that TMC1 and TMC2 are not wholly equivalent in mammals (Nakanishi et al., 2018), and this may be true for zebrafish Tmc protein isoforms as well.

To examine the fundamentals of Tmc function in hearing, we used the saccule, the primary hearing organ of the larval zebrafish ear, and the *tmc2b*^cwr2^ *tmc2a*^cwr3^ double mutant zebrafish, which has just a handful of hair cells that permit attenuated hearing. This simplified genetic *tmc* background has a single Tmc protein, Tmc1, that enables inconsistent hearing as we described in Chen et al. (2020). More precisely, in *tmc2b*^cwr2^ *tmc2a*^cwr3^ double mutants, when presented with vibrational stimuli, just 39% were reacted to with a C-start reflex; in contrast, controls reacted to 100% of stimuli (Chen et al., 2020). The variability in responsiveness occurred within individual fish. By mapping hair cell position, hair bundle orientation, and the functional hair cells of the saccule, we identify a set of succinct rules for Tmc1 function and ultimately hearing. One, hair cell location is a key factor that correlates with Tmc1 dependence. Two, Tmc1-dependent hair cells occupy a single axis of best sensitivity. Three, not all hair cells depend on Tmc1 in this axis. Four, hair bundles that depend on Tmc1 face both directions within the axis of best sensitivity and are equally distributed along a mirror plane of symmetry (Supplementary Figure S1). Other factors that may correlate with hair cell Tmc dependency, such as hair cell innervation pattern and the ultimate destination within the central nervous system the mechanical information derived from a particular Tmc is sent, are potential areas that may reveal the significance of Tmc diversity.

Finally, these data suggest minimal parameters for hearing in a vertebrate. This is because *tmc2b*^cwr2^ *tmc2a*^cwr3^ double mutant zebrafish can hear (Chen et al., 2020), and the saccule is the major hearing organ of larvae (Inoue et al., 2013). Hearing can occur with just 10–18 hair cells functional in each saccule. These cells need to only express Tmc1 and lay within a single (dorsal-ventral) axis of best sensitivity (Figure 4B). Therefore, hair cells within the anterior–posterior axis of best sensitivity are not required to elicit a startle response. In summary, by investigating Tmc1’s role in the saccule, we have established minimal anatomical and molecular genetic requirements and relationships for hearing.

## Methods

### Zebrafish

Protocols for housing and handling of zebrafish were approved by Case Western Reserve University’s Institutional Animal Care and Use Committee. Wild-type *Tübingen* (*Tü*), *Tg(myo6b:GCaMP6s-caax)* transgenic (Lukasz and Kindt, 2018), *tmc1*^cwr5^ mutant, *tmc2a*^cwr3^ mutant, *tmc2b*^cwr2^ *tmc2a*^cwr3^ double mutant, and *tmc2b*^cwr8^ *tmc1^cwr4^ tmc2a^cwr6^* triple mutant fish (Chen et al., 2020) were used in this study.

### Mutation of *tmc1* and *tmc2a* With CRISPR/Cas9

CRISPR gene editing was performed as described previously (Chen et al., 2020). *Tmc1* (ENSDARG00000056386) was targeted with an sgRNA complementary to exon 7 (5′-TGGGCTG GTCATGGTTCCAG-3′). *Tmc2a* (ENSDARG00000033104) was targeted with an sgRNA complementary to exon 9 (5′-AGGTC CCAATGCCCACCATG-3′). The mutations in each gene carried by founder fish and F1 fish were identified using sequencing primers described previously (Chen et al., 2020). *Tmc1*^cwr4^ mutant fish with an 8-bp deletion and *tmc2a*^cwr7^ mutant fish with a 1-bp indel were used for assays in this work. The *tmc2a*^cwr7^ single mutant fish was isolated from a crossover recombination event after mating *tmc1*^cwr4/+^ *tmc2a*^cwr7/+^ fish with wild-type animals and subsequent incrossing.

### Transgenesis

To generate *Tg(myo6b:Cerulean)* zebrafish, DNA plasmid, pMT/myo6b/cerulean, at 100 ng/μl and *Tol2* RNA at 62.5 ng/μl were mixed with phenol red and 0.125 mM KCl solution and then coinjected into *Tü* zebrafish embryos at the one-cell stage to create somatic transgenics. To generate a stable transgenic line, positive founder fish were outcrossed with *Tü* zebrafish.

To generate *Tg(myo6b:GCaMP6s-CAAX)*; *tmc2b*^cwr2^ *tmc2a*^cwr3^ zebrafish, we crossed *tmc2b*^cwr2/+^ *tmc2a*^cwr3^ fish with stable transgenic *Tg(myo6b:GCaMP6s-CAAX)* fish. We then crossed *Tg(myo6b:GCaMP6s-CAAX)*; *tmc2b*^cwr2/+^ *tmc2a*^cwr3/+^ fish with *tmc2b*^cwr2/+^ *tmc2a*^cwr3^ fish to obtain *Tg(myo6b:GCaMP6s-CAAX)*; *tmc2b*^cwr2^ *tmc2a*^cwr3^ zebrafish. Crossing was used to generate *Tg(myo6b:GCaMP6s-CAAX)*; *tmc1*^cwr4^ zebrafish.

### Fixed and Live Imaging

Fixation and FM1-43FX imaging were performed as described previously (Chou et al., 2017). For live imaging, the larvae were anesthetized in 0.612 mM ethyl 3-aminobenzoate methanesulfonic acid (Sigma-Aldrich) in fish water and mounted laterally on glass-bottom dishes (MatTek) in 1.0% (wt/vol) low-melting-point agarose (Promega). Z-stack images of the entire lateral crista were collected on a confocal microscope (TCS SP8, Leica) with a 40 × /1.3 NA oil-immersion objective. A laser wavelength of 405 nm was used for Cerulean excitation. A laser wavelength of 488 nm was used for 4-Di-2-ASP excitation. Laser wavelengths of 545 and 488 nm were used for FM4-64 and GCaMP6s excitation, respectively. For the identification of hair bundle orientations, Alexa Fluor 633 phalloidin at 1:50 dilution was used to label actin filaments (Chou et al., 2017; Chen et al., 2020). For Alexa Fluor 633, an excitation wavelength of 633 nm was used.

### Image Processing and Quantification

Confocal images were imported to and analyzed in ImageJ (NIH). A Gaussian blur filter (radius = 1.00) was applied to all images to reduce random noise. For Cerulean, GCaMP6s, and phalloidin channels, the brightness/contrast were adjusted automatically to enhance the morphological appearance of hair cells or hair bundles. For the styryl dye (4-Di-2-ASP, FM4-64, and FM1-43FX) emission channels, raw data are presented. Images of partial Z-stack maximum projections are shown for the lateral crista to minimize hair cell overlap. For mapping, images of whole Z-stack maximum projections are shown for the posterior macula.

Due to the densely overlapping nature of crista hair cells, cell length measurements or enumeration was performed manually by scanning the Z-stack series of Cerulean-labeled or FM4-64/GCaMP6s double-labeled hair cells, respectively. Graphs were generated in GraphPad Prism version 8 (GraphPad Software).

### Statistics

All statistics were performed by GraphPad Prism version 8. Data are reported as mean ± SEM or SD. Comparisons between groups were tested by one-way ANOVA or Student’s *t*-test. Short hair cells were judged to be positive for mechanotransduction if their fluorescence intensity was 2.33 standard deviations greater than the background intensity (Honegger et al., 2011).

### Quantification of FM4-64 Intensity

To quantitate FM4-64 uptake in the lateral crista hair cells, a 0.75 μm^2^ square region of interest (ROI) was defined manually within the hair cell, not overlapping with other hair cells, using the GCaMP6s-CAAX images taken simultaneously (LAS X, Leica). Each ROI selected for quantitation was from the optical slice with the maximum fluorescence intensity of that hair cell’s Z-stack. After background subtraction, the average of the FM4-64 intensities of individual hair cells in the lateral crista were normalized to the average of the intensity of the corresponding control group (Figure 2C).

## Data Availability Statement

The raw data supporting the conclusions of this article will be made available by the authors, without undue reservation.

## Ethics Statement

The animal study was reviewed and approved by IACUC.

## Author Contributions

SZ carried out gene editing. SZ and HW performed the dye injection. ZC and SZ performed the imaging. BM wrote the manuscript. All authors developed the experimental plans and commented on and contributed to the final version of the manuscript.

## Conflict of Interest

The authors declare that the research was conducted in the absence of any commercial or financial relationships that could be construed as a potential conflict of interest.

## Supplementary Material

Supplementary Figure 1 Symmetric and asymmetric models of Tmc dependencies within the ear.
