# Supplementary material for: Tmc Reliance Is Biased by the Hair Cell Subtype and Position Within the Ear
Source: Front Cell Dev Biol. 2021 Jan 7;8:570486. doi: 10.3389/fcell.2020.570486 (PMC7817542; doi:10.3389/fcell.2020.570486)
Supplement: Supplementary file 1 [file Table_1.docx]

**Supplementary table 1.** Genotypes

| **Gene** | **Allele** | **Exon** | **Type** | **Target (5'->3')** | **Genotype** | **DNA change** | **aa preserved/ total aa** | **DNA change description** |
| --- | --- | --- | --- | --- | --- | --- | --- | --- |
| ***tmc1*** | cwr4 | 7 | small deletion | TGGGCTGGTCATGGTTCCAG | *tmc1 ^cwr4^* | 8-bp deleted | 385 / 990 | -8 bp at c.1155-1162  (-GAACCATG) |
|  | cwr5 | 5 | small deletion | Left: TTGTGGAGAAATATGAAG; Right: CAAAGGCAGGAAACTTTA | *tmc1 ^cwr5^* | 1-bp deleted | 308 / 990 | -1 bp at c.926  (-T) |
| ***tmc2a*** | cwr3 | 9 | small deletion | AGGTCCCAATGCCCACCATG | *tmc2a ^cwr3^* | 1-bp deleted/  1-bp deleted | 349 / 916 | -1 bp at c.1050 (-C);  -1 bp at c.1052 (-T) |
|  | cwr6 |  | indel |  | *tmc2a ^cwr6^* | 2-bp inserted/  3-bp deleted | 350 / 916 | -3/+2 bp at c.1053-1055 (-GGT/+TG) |
|  | cwr7 |  | indel |  | *tmc2a ^cwr7^* | 2-bp inserted/  1-bp deleted | 350 / 916 | -1/+2 bp at c1052  (-T/+GA) |
| ***tmc2b*** | cwr2 | 7 | small deletion | GGCCGAATCCTGCTCCTCTC | *tmc2b ^cwr2^* | 5-bp deleted | 266 / 892 | -5 bp at c.799-703  (-GAGGA) |
|  | cwr8 | 6 | small deletion | GGACTAAACCTTGTTCTCTT | *tmc2b ^cwr8^* | 5-bp deleted | 236 / 892 | -5 bp at c.710-714  (-TCTTC) |
