# Supplementary material for: Tmc Reliance Is Biased by the Hair Cell Subtype and Position Within the Ear
Source: Front Cell Dev Biol. 2021 Jan 7;8:570486. doi: 10.3389/fcell.2020.570486 (PMC7817542; doi:10.3389/fcell.2020.570486)
Supplement: Supplementary file 2 [file Table_2.docx]

**Supplementary table 2.** Mutants

| **Mutant fish** | **Allele** | **Putative Zebrafish International Resource Center (ZIRC) name** |
| --- | --- | --- |
| ***tmc1* single mutants** | cwr4 | *tmc1 ^cwr4^* |
|  | cwr5 | *tmc1 ^cwr5^* |
| ***tmc2a* single mutant** | cwr7 | *tmc2a ^cwr7^* |
| ***tmc2a* single mutant** | cwr3 | *tmc2a ^cwr3^* |
| ***tmc1* *tmc2a* double mutant** | cwr4 cwr7 | *tmc1 ^cwr4^ tmc2a ^cwr7^* |
| ***tmc2b* *tmc2a* double mutant** | cwr2 cwr3 | *tmc2b ^cwr2^ tmc2a ^cwr3^* |
| ***tmc2b* *tmc1* *tmc2a* triple mutant** | cwr8 cwr4 cwr6 | *tmc2b ^cwr8^ tmc1 ^cwr4^ tmc2a ^cwr6^* |
