# Supplementary figures and images for: Tmc Reliance Is Biased by the Hair Cell Subtype and Position Within the Ear
Source: Front Cell Dev Biol. 2021 Jan 7;8:570486. doi: 10.3389/fcell.2020.570486 (PMC7817542; doi:10.3389/fcell.2020.570486)

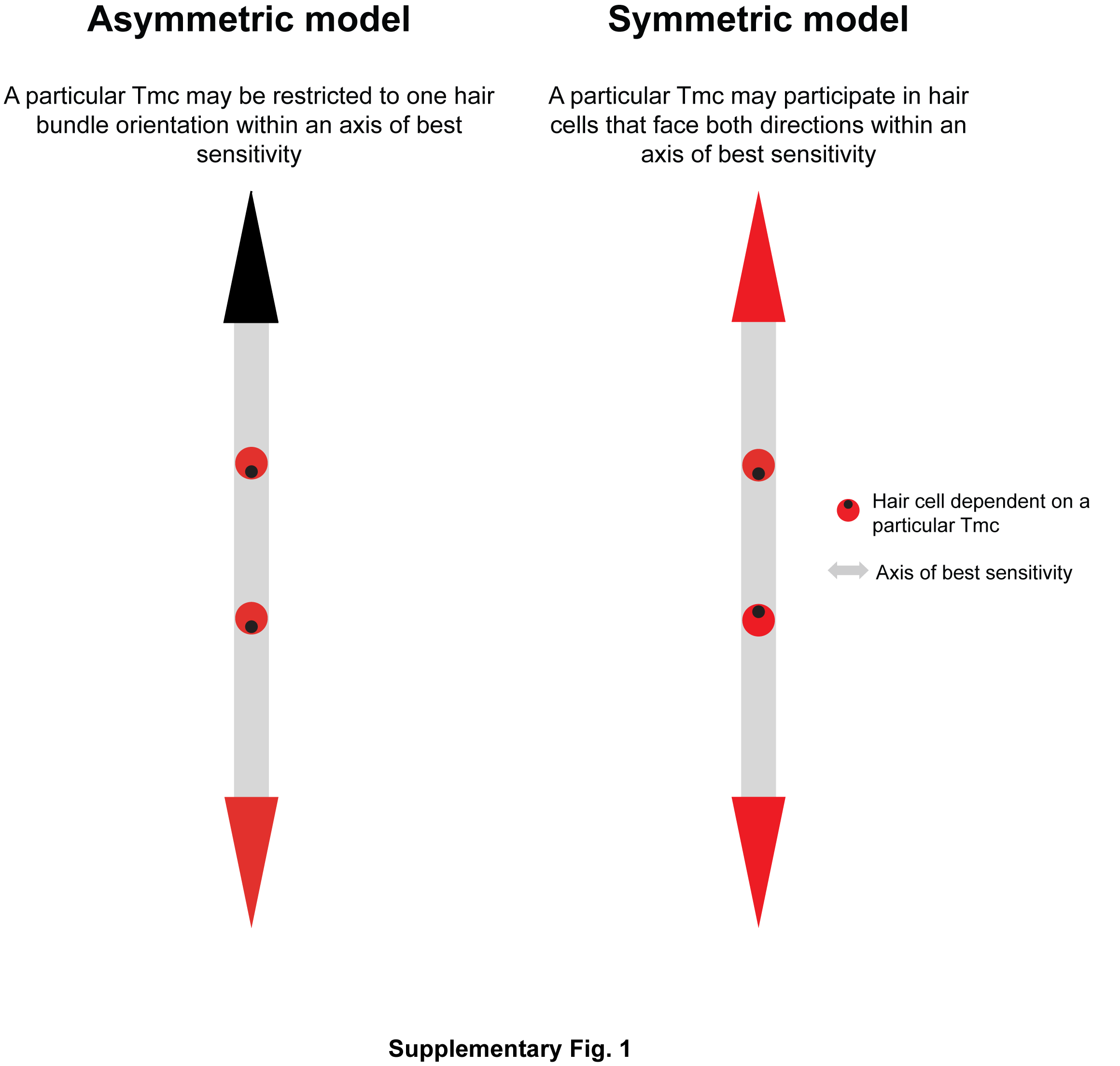

Supplement: Supplementary Figure 1 — Symmetric and asymmetric models of Tmc dependencies within the ear. [file Image_1.tif]
